# Fecal carriage of ESBL-producing *E. coli* and genetic characterization in rural children and livestock in the Somali region, Ethiopia: a one health approach

**DOI:** 10.1186/s13756-024-01502-5

**Published:** 2024-12-18

**Authors:** Abdifatah Muhummed, Ashenafi Alemu, Salome Hosch, Yahya Osman, Rea Tschopp, Simon Yersin, Tobias Schindler, Jan Hattendorf, Jakob Zinsstag, Guéladio Cissé, Pascale Vonaesch

**Affiliations:** 1 Swiss Tropical and Public Health Institute, Kreuzstrasse 2, 4123 Allschwil, Switzerland; 2 University of Basel, Petersplatz 1, 4003 Basel, Switzerland; 3 Present Address: Department of Fundamental Microbiology, University of Lausanne, UNIL-Sorge, 1015 Lausanne, Switzerland; 4 Jigjiga University, Jigjiga, Ethiopia; 5 Armauer Hansen Research Institute, PO Box 1005, Addis Ababa, Ethiopia

**Keywords:** Antimicrobial resistance, Extended spectrum beta lactamase, *E. coli*, One Health

## Abstract

**Background:**

The emergence and spread of Extended-Spectrum Beta-Lactamase (ESBL)-producing *Escherichia coli* pose significant challenges for treatment of infections globally. This challenge is exacerbated in sub-Saharan African countries, where the prevalence of ESBL-producing *E. coli* is high. This, combined with the lack of a strong and supportive healthcare system, leads to increased morbidity and mortality due to treatment failures. Notably, studies in Ethiopia have primarily focused on hospital settings, leaving a gap in understanding ESBL prevalence in rural communities, where human-animal proximity may facilitate microbial exchange.

**Methods:**

We conducted a community-based study in the rural Somali region of Ethiopia, simultaneously examining the fecal carriage of ESBL-producing *E. coli* in children aged 2–5 years and their livestock (cattle, camel, goat). Fecal samples from 366 children and 243 animals underwent phenotypic screening for ESBL-producing *E. coli*. Following phenotypic confirmation, ESBL resistance genes were identified via conventional PCR. Whole-genome sequencing (WGS) was performed on a subset of isolates from human feces.

**Results:**

We found that 43% (159/366) of children and 3.7% (9/244) of livestock harbored ESBL-producing *E. coli*. The ESBL gene *bla*_CTX-M-15_ was predominant in human (82.7%, 120/145) and livestock (100%) isolates. In the 48 human *E. coli* isolates subjected to WGS, a high diversity resulting in 40 sequence types (STs) was observed. Among these, ST-2353 was the most prevalent (5/48), followed by ST-10 and ST-48 (3/48) and ST-38, ST-450, and ST-4750 (2/48). These STs were associated with multiple resistance genes, such as *bla*_CTX-M-15_, *bla*_TEM-1B_, *bla*_OXA-1_, *bla*_CTX-M-14_ and *bla*_TEM-35_.

**Conclusion:**

We report a high prevalence of ESBL *E. coli* in rural children, which outnumbers its prevalence in livestock. These isolates displayed a high diversity of sequence types (STs) with ST-2353 being the dominant ST. Our study is the first to report the association of ST-2353 with multi-drug resistance genes in Ethiopia. Further research using an integrated approach including other domains such as water and food products is needed to truly understand and combat AMR transmission and acquisition in this region.

**Supplementary Information:**

The online version contains supplementary material available at 10.1186/s13756-024-01502-5.

## Introduction

The emergence and spread of multidrug-resistant bacterial strains, particularly Extended-Spectrum Beta-Lactamase (ESBL)-producing *Escherichia coli* (*E. coli*), pose significant challenges for treatment of infections globally [1]. These are exacerbated in sub-Saharan African countries, including Nigeria, Tanzania and Ghana, where the prevalence of ESBL-producing *E. coli* is high, both in humans (up to 60%) and animals (up to 56%) [2–6]. This, combined with the lack of a strong and supportive healthcare system, leads to increased morbidity and mortality due to treatment failures [7]. Robust laboratory diagnostics and strong surveillance systems for antimicrobial resistance are urgently needed, yet, they are still lacking in many parts of sub-Saharan Africa [8].

ESBLs are enzymes that hydrolyze a broad range of beta-lactam antibiotics, confering resistance to multiple antibiotics [9]. CTX-M, a dominant ESBL type, has become widespread globally after the late 1990s, outpacing TEM and SHV [10]. Nowadays, its prevalence is high in infections of humans and food-producing animals, and it is considered one of the main contributing factors to multidrug resistance in *E. coli* in both low- and middle-income (LMICs) and Western countries [11–13].

ESBL genes, commonly found in plasmids, primarily spread through horizontal gene transfer [14, 15]. Recent findings suggest they can also exist in the chromosome, indicating potential clonal transmission [16, 17]. Transmission of ESBL-producing *E. coli* from animals to humans, often discussed in the context of contaminated animal-origin food, is a concern [18]. Surveillance relies on identifying similar clones, plasmids, or sequence types in human and animal populations, to infer transmission [19]. The interconnectedness of infections in both species underscores the need for a One Health approach to fully understand the dynamics and spread of resistant clones.

In Ethiopia, recent studies have revealed a high prevalence of *bla*_*CTX-M*_, especially the *bla*_CTX-M-15_ variant (87.7–88.4%), within hospital settings, which also conferred resistance to non-beta-lactam antibiotics, such as fluoroquinolones and aminoglycosides [20–22]. Additionally, these studies reported different *E. coli* phylo-groups, with several isolates linked to the ST-131 clone, which is mainly associated with *bla*_CTX-M-15_ [20–22]. Notably, studies in Ethiopia have primarily focused on hospital settings, leaving a gap in understanding ESBL prevalence in rural communities, where human-animal proximity may facilitate microbial exchange.

To fill this gap, we conducted a community-based study to investigate the fecal carriage of ESBL-producing *E. coli* in children aged two to five years and livestock of the same households and genetically characterized the isolated clones in the feces of children and livestock. By identifying the prevalence of ESBL-producing *E. coli* and their genetic characteristics, this study yields new insights to guide interventions aimed at curbing the dissemination of antimicrobial resistance and enhancing treatment strategies for infectious diseases caused by these bacteria.

## Methods

### Study area

The study was carried out from May 2021 until June 2021 in Adadle district in the Shebele zone, Somali Regional State (SRS), Ethiopia.

### Sample size

Based on a previous study conducted in Addis Ababa, we expected the prevalence of ESBL-producing *E. coli* to be 42% [23] and assumed an inter-cluster correlation coefficient of 0.15. We calculated a sample size of 360 eligible children, with 180 children from pastoralist and 180 children from agro-pastoralist communities to be sufficient to estimate the prevalence with a margin of error of 10% at the 95% confidence level.

### Selection criteria

Based on the nature of livelihood, eight of the thirteen Kebele in the Adadle district were randomly selected. Among the selected Kebele, four were pastoralist (Malkasalah, Todab, Harsug, Boholhagere) and four were agro-pastoralist (Bursaredo, Dabafyd, Gabal, Higlo).

A pre-enrolment screening was carried out in each Kebele to assess children between 2 and 5 years of age. All children were screened for stunting and wasting based on WHO guidelines. All stunted (height for age score, HAZ <− 2), and wasted (weight for height score, WHZ <− 2 or mid-upper arm circumference (MUAC) < 11.5 cm) were enrolled in the study. Among the screened children who were non-stunted and/or non-wasted, a random selection process was employed from an Excel file containing information on all pre-screened households/children in the community. The random selection process continued until the desired sample size was achieved. Children outside the age range (below 2 years and above 5 years) and those who had received antibiotic treatment within the last 14 days were excluded from the study. Informed consent was obtained from all participants’ legal guardians.

### Anthropometric measurements

Height measurements were taken with children standing against a WHO standard wooden measuring board, ensuring the correct posture and position. Weights were recorded using a WHO standard weight scale, either the child alone or with the mother by adjusting the weight scale [24]. MUAC measurements were performed using a WHO standard tape measure. To ensure the accuracy of height, weight and MUAC measurements, each anthropometric measurement was repeated at least twice or until the measurements were within 1 mm, 100 g, or 1 mm of one another, respectively.

### Data collection

A comprehensive questionnaire was administered to the mother of the child by trained field workers in the local language using Open Data Kit (ODK). The questionnaire covered different sections, including demographic characteristics, child health status, WASH behavior, breastfeeding, diet, and anthropometric measurements. As birth records were not available in the community, the child’s age was estimated based on group discussion involving the child’s mother, as well as other family and community members. These discussions considered factors such as seasonal events (floods, drought, and rainy season) and festive occasions (Ramadan, festival, and other religious events) that occurred before and after the child’s birth. Additionally, leveraging the close-knit relationships within the community, efforts were made to engage in discussions to collectively recollect and remind each other about significant events and timeframes related to the child’s birth.

### Stool sample collection

Trained field workers provided mothers with instructions in the local language a day before collecting fecal samples. Mothers used sterile specimen cups, and upon receipt, samples were placed in an ice box and transported within 2–4 h to a field laboratory. There, samples were aliquoted into six cryotubes, two with glycerol (40% glycerol mixed 1:1 with the stool) and four without, stored at 4 °C. Subsequently, samples were sent to Gode within 24 h, stored at − 20 °C, and then shipped to the Armauer Hansen Research Institute in Addis Ababa, where they were preserved at − 80 °C for long-term storage.

Additionally, from the selected households that owned animals, we assessed different types of animals (camels, cattle, and goats). We randomly sampled at least one animal per species. Animal fecal samples were collected by a veterinary professional through either rectal insertion of gloved hands or aseptic collection of fresh stool (if the animals defecated in the presence of the team). Animal samples underwent a process similar to human samples—immediate shipment to the field laboratory, aliquoting, storage at − 20 °C, and final storage in Addis Ababa at − 80 °C for future analysis.

### Bacterial isolation and identification

Fecal samples were aseptically inoculated onto MacConkey agar plates (Oxoid, UK) [25], and subsequently, a cefotaxime disc was placed at the center of the plate. The plates were then incubated for 24 h at 37 °C. The *E. coli* colony closest to the cefotaxime disc was meticulously selected based on its distinctive morphology and pigmentation characteristics. The selected colonies were re-inoculated overnight to ensure purity and, and subsequently, the colony was confirmed as *E. coli* through a biochemical test. Isolates were classified as *E. coli* if they exhibited a positive indole test, negative citrate, positive lysine decarboxylation, gas and acid production, mannitol fermentation, negative urea hydrolysis, and if they were motile.

### Antibiotic susceptibility test

The disc diffusion method, was used to determine the antibiotic susceptibility using Müller-Hinton agar (Oxoid, UK) according to the recommendation of the Clinical and Laboratory Standard Institute (CLSI) guidelines [26]. Antibiotic susceptibility tests were performed for 19 antibiotics from 10 different classes, including penicillins (ampicillin (AMP) 10 µg and amoxicillin (AML) 10 μg), beta-lactamase inhibitor combinations (ampicillin-sulbactum (SAM) 30 μg and amoxicillin/clavulanic acid (AMC) 30 µg), cephalosporin (cefazolin (KZ) 30 µg, cefpodoxime (CPD) 10 µg, cefuroxime (CXM) 30 µg, ceftriaxone (CRO) 30 µg, cefotaxime (CTX) 30 µg, cefipime (FEP) 30 µg, ceftazidime (CAZ) 30 µg), carbapenem (imipenem (IPM) 10 µg and ertapenem (ETP) 10 µg), monobactams (aztreonam (ATM) 30 µg), fluoroquinolones (ciprofloxacin (CIP) 5 µg), aminoglycosides (gentamicin (CN) 30 µg), macrolides (azithromycin (AZM) 15 µg), tetracycline (TE) 30 µg), and cotrimoxazole (SXT) 25 µg (Oxoid, United Kingdom). McFarland standard 0.5 was used prior to spreading the suspension on the Müller-Hinton agar plate to ensure the bacterial suspension turbidity. Plates were incubated at 37 °C for 18 h. Based on the inhibition zone diameters, antibiotics were assigned to susceptible, intermediate, and resistant as indicated by CLSI guidelines [26]. Multidrug resistance was defined as non-susceptible to at least one antibiotic agent in three or more antimicrobial classes [27]. Extensive drug resistance (XDR) was defined as resistance to at least one agent in all but susceptible to two or fewer antimicrobial categories. Pan drug resistance (PDR) was defined as resistance to all agents in all antimicrobial categories [28].

### ESBL screening and confirmatory test

According to CLSI guidelines, any strain showing resistance against cefotaxime 30 μg, ceftazidime 30 μg, or ceftriaxone 30 µg was considered as a potential ESBL producer. Confirmatory tests were performed utilizing a disc of cefotaxime 30 μg and one of ceftazidime 30 μg alone, and a disc of cefotaxime and one of ceftazidime combined with clavulanic acid 10 µg (Oxoid, United Kingdom). Isolated strains were considered ESBL-producers if an increase of inhibition zone diameter of 5 mm or greater was observed in the discs of cefotaxime or ceftazidime combined with clavulanic acid compared to the inhibition observed in the cefotaxime or ceftazidime discs without clavulanic acid. *E. coli* ATCC 25922 and *E. coli* ATCC BAA-2326 were used as negative and positive controls, respectively [29].

### Molecular testing for β-lactamase genes (*bla*_CTX-M_*, bla*_TEM_*, and bla*_SHV_)

The DNA of the confirmed ESBL-producing isolates was extracted using the Wizard^®^ HMW DNA Extraction Kit, according to the manufacturer’s instructions [30]. The presence of three ESBL genes (*blaCTX-M, blaTEM, and blaSHV*) was detected using a multiplex PCR approach within a single reaction tube. Details regarding the PCR reaction, cycling, and primers for amplifying the ESBL resistance genes can be found in supplementary materials 1.

### Whole genome sequencing

The extracted DNA from human and animal ESBL-producing isolates were shipped to the Swiss Tropical and Public Health Institute (Swiss TPH) for whole genome sequencing (WGS). However, the DNA quality of the sub-isolates, selected based on their phenotypic profiles, was insufficient for WGS. Since we did not have the original isolates in Switzerland, we decided to re-culture and perform DNA extraction on the original fecal samples that were initially shipped with the DNA. Subsequently, 48 of these samples were selected based on the phenotypic profiles obtained in Ethiopia and the phenotypic analysis was repeated for the new isolates for the data provided in Fig. 4.

DNA concentration was quantified with the Qubit dsDNA HS Assay Kit (Invitrogen, Germany). Isolates were selected for WGS using the MinION platform (Oxford Nanopore Technologies, UK) based on DNA concentrations (> 33 ng/µL). The sequencing library was prepared according to the manufacturer’s instructions using the Native Barcoding Kit 96 (SQK-LSK114.96) and loaded onto the R10.4.1 flow cell and sequenced on the MinION Mk1C using super-accurate base calling.

### Bioinformatics analysis

De novo assembly was conducted using Flye 2.9.1 at the scientific computing core facility of the University of Basel [31]. The Bacterial and Viral Bioinformatics Resource Center (BV-BRC) was used for annotation and phylogenetic analysis of the assemblies. The assemblies were annotated using the RAST 2.0 toolkit [32]. Only assemblies with less than 20 contigs were considered for further analysis. Sequence type of assembled contigs were determined using MLST [33]. Phylogenetic trees were generated by aligning protein and nucleotide sequences using MUSCLE, MAFFT and RAxML [33–35]. Resistome analysis was performed using CARD (v. 6.0.0) and ResFinder (v. 4.2.2) [34, 36]. VirulenceFinder v.2.0 and PlasmidFinder v.2.1 tools available at the Center for Genomic Epidemiology were used for the virulent detection and plasmid replicon typing [37].

### Statistical analysis

R statistical software v. 4.1.3 was used to perform the statistical analysis [38]. Initial descriptive analysis of variables was performed using the gtsummary package. Multivariate analysis employed logistic regression to assess the association between ESBL-producing *E. coli* and independent variables. The initial model included variables (age, sex, education, sanitation practice, hygiene, source of water, owning livestock, nutritional status) based on literature knowledge, with stepwise removal of those contributing insufficient information (p > 0.2). Variables with p < 0.2 were retained. Model fitness was assessed using likelihood ratio test, AIC (Akaike Information Criterion), and adjusted R square. Significance was determined at p < 0.05. R packages ape, ggplot2, ggtree, and ggtreeExtra were used for analyzing resistance genes and visualizing phylogenetic trees.

## Results

### Description of study population

Out of the 366 children, only 346 children completed the questionnaire and were included in this study for further analysis. Pastoralists and agro-pastoralists were evenly represented in terms of age and sex among the enrolled children. Over half of the children showed a normal growth (61%), while a quarter (25%) of them were wasted, and 7.8% were stunted. Further, 5.2% of the children suffered concomitantly from stunting and wasting. Characteristics of the study group are summarized in Table 1.

**Table 1:** Characteristic of pastoralist and agro-pastoralist in the Adadle district, Somali region, Ethiopia

Variables	N = 346 (%)
Settlement area	
Pastoralist	177 (51.2%)
Agro-Pastoralist	169 (48.8%)
Education of the mothers	
Formal education	50 (14.5%)
Non-formal education	36 (10.4%)
Illiterate	260 (75.1%)
Sex of the child	
Female	176 (50.9%)
Male	170 (49.1%)
Age group of the child	
2 years	150 (43.4%)
3 years	98 (28.3%)
4–5 years	98 (28.3%)
Number of children per household	
1–3 children	78 (22.5%)
4–6 children	160 (46.2%)
> = 7 children	108 (31.2%)
Completed all required vaccinations	
No	309 (89.3%)
Yes	37 (10.7%)
Has a vaccination card	
No	328 (94.8%)
Yes	18 (5.2%)
Nutritional status	
Normal growth	212 (61.3%)
Wasted	88 (25.4%)
Stunted	27 (7.8%)
Stunted and wasted	18 (5.2%)
Overweight	1 (0.3%)

As summarized in the suppmentary Table S1, the two main sources of water for pastoralists and agro-pastoralists were rainwater/birkad (89%) and river water (95%), respectively. Most of pastoralist and agro-pastoralist (96%) used open space for defecation. The 14 households (4%) that had a toilet shared it with 28 households. Dumping waste in the street or open spaces within the compound was the predominant method of waste disposal in both pastoralist and agro-pastoralist societies (84%), while 16% opted to burn waste. Most mothers reported that they used water to wash the children’s hands (93%), while a small subfraction of mothers reported (4%) using water and soap. At the time of the sampling, more than half of the agro-pastoralists and pastoralists had soap, while 29% did not have soap very often and 13% never had soap in their house.

Nearly half of agro-pastoralist households (43%) and 12% of pastoralist households treated the water prior to consumption (mainly chlorination (98.9%)). The majority of agro-pastoralists possessed cattle (94.7%), donkeys (81.1%), goats (62.7%), sheep (53.3%), camels (10%) and chickens (10%). Among pastoralists, predominant livestock ownership included goats (87.6%), donkeys (58.8%), sheep (44.1%), camels (36.2%), and cattle (29.9%).

### Phenotypic test results for ESBL *E. coli* carriage

A total of 609 fecal samples, comprising 366 from humans and 243 from animals (including 77 goats, 136 cows, and 30 camels) were analysed. For human isolates, 159 (43%) of the *E. coli* isolates were ESBL-producers (24.5% in pastoralists and 18.8% in agropastoralists). Furthermore, 7.8% of the *E. coli* isolates from goats (6/77) and 2.2% from cows (3/136) were ESBL-producers. Regarding animal ESBL-producing isolates, 7 (77.8%) were identified among animals of agro-pastoralists, while 2 (22.2%) were found among those of pastoralists.

### Susceptibility pattern for ESBL-producing *E. coli*

For the human isolates, ESBL-producing *E. coli* strains exhibited complete resistance to several commonly used antibiotics including amoxicillin, cefotaxime, cefuroxime, ceftriaxone, cefazolin, and cefpodoxime. Almost all ESBL-producing *E. coli* isolates (98.7%) were resistant to ampicillin and 51.6% were resistant to tetracycline.

Regarding co-trimoxazole, 57.9% of the isolates were classified as resistant, 2.5% as intermediate, and 37.7% as susceptible. Among ESBL-producing *E. coli*, 47.8%, 42.1%, and 27.7% were non-susceptible, and 32.1%, 54.7%, and 25.2% were intermediate to aztreonam, cefipime, and ceftazidime, respectively. In addition, 15.7% and 12.6% of the isolates were resistant to azithromycin and ciprofloxacin. Ampicillin-sulbactam (8.8%), gentamicin (6.9%), and amoxicillin-clavulanic acid (5.9%) had the lowest rates of resistance. Intermediate susceptibility was observed in 16.5% for amoxicillin-clavulanic acid, 15.1% for ampicillin-salbactum, and 2.5% for gentamicin. All carbapenems (imipenem and ertapenem), which are considered the last-resort antibiotics for infections caused by ESBL-producing *E. coli*, were found to be effective against the ESBL-producing *E. coli* isolates, except for 1.3% of the isolates that had intermediate susceptibility to ertapenem. Additionally, using whole genome sequencing (WGS), none known carbapenem or colistin resistance genes were found in the *E. coli* isolates.

**Fig. 1:**
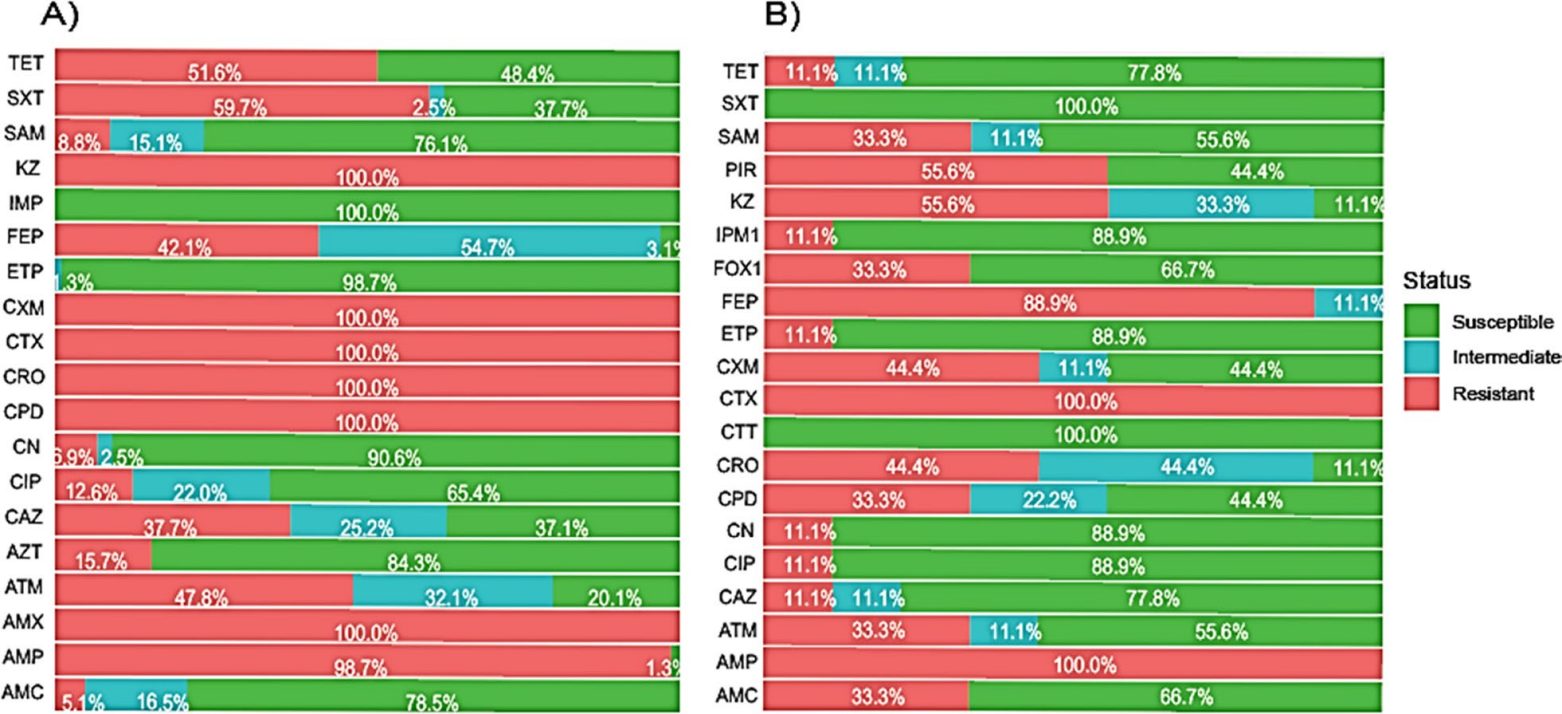
Antimicrobial resistance pattern of ESBL-producing *E. coli* isolated from the feces of children aged 2–5 years (**A**, 159/366) and from livestock (**B**, 9/243) in the Adadle district, Somali region, Ethiopia. Antimicrobial agents tested include: AMX (Amoxicillin), AMC (Amoxicillin-clavulanic acid), AMP (Ampicillin), ATM (Aztreonam), AZT (Azithromycin), CAZ (Ceftazidme), CIP (Ciprofloxacin), CN (Gentamicin), CPD (Cefpodoxime), CRO (Ceftriaxone), CTX (Cefotaxime), CCT (Cefotetan), ETP (Ertapenem), FEP (Cefepime), FOX (Cefoxitin), IMP (Imipenem), KZ (Cefazolin), SAM (*Ampicillin-sulbactam)*, SXT (Co-trimoxazole), PIR (Piperacillin), TET (Tetracycline)

Additonally, nine ESBL isolates obtained from animal fecal samples exhibited complete (100%) resistance to ampicillin, cefotaxime, and amoxicillin. Furthermore, an 88.9% resistance rate was observed for cefepime, followed by cefazolin (55.6%), ceftriaxone (44.4%), cefuroxime (44.4%), and aztreonam and ampicillin-sulbactam, which both exhibited an equal resistance rate of 33.3%. In ESBL-producing *E. coli* isolates from animals, ampicillin-clavulanic acid, ceftazidime, gentamicin, and ciprofloxacin all exhibit an equal resistance rate of 11.1%. The data is summarized in Fig. 1.

All ESBL-producing *E. coli* strains exhibited multi-drug resistance (MDR), rendering them non-susceptible to three or more antibiotic drug classes (Figure S1 in Supplementary File 1).

Thus, ESBL-carriage is very prevalent, especially in children, in a community setting and is much lower in the feces of livestock animals.

### Predictors of ESBL-producing *E. coli* carriage

**Fig. 2:**
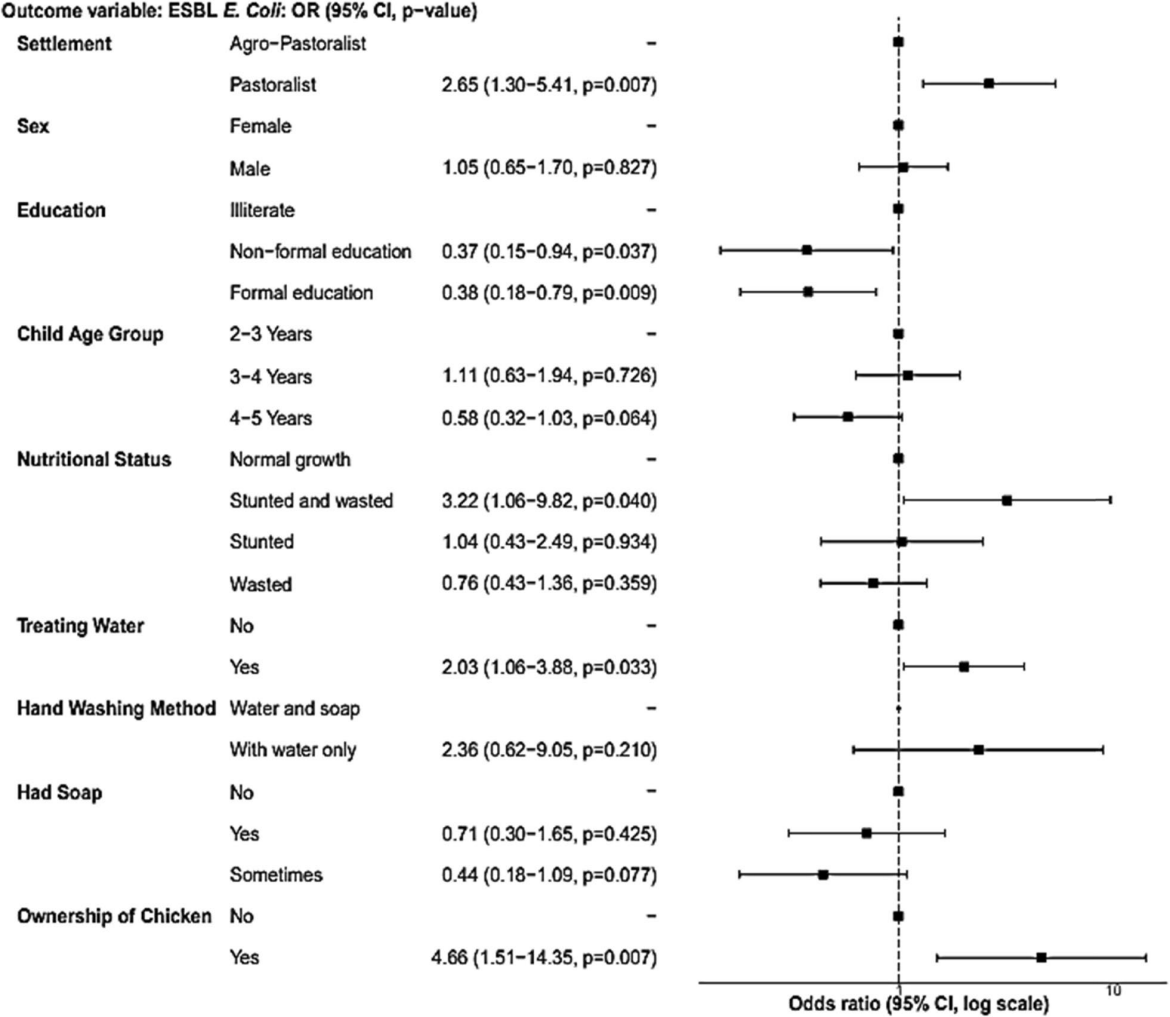
Risk factors (multivariable model) associated with fecal carriage of ESBL-producing *E. coli* among children living in the Adadle district, Somali region, Ethiopia

In the multivariable analysis, education was significantly associated with ESBL-producing *E. coli*. Children whose mothers or household heads were illiterate had twice the odds of carrying ESBL-producing *E. coli* compared to children whose mothers were formally educated (aOR = 2.65, 95% CI = 1.27–5.48). The odds of children who were both stunted and wasted were three time higher to harbor ESBL-producing *E. coli* compared to children with normal growth (aOR = 3.14, 95%CI = 1.02–9.07). Moreover, pastoralist children had 2.65 times higher odds of being colonized with ESBL producing *E. coli* compared to agro-pastoralist children (aOR = 2.65, 95% CI = 1.30–5.41). Counterintuitively, children who drank water treated with chlorine showed a positive association with ESBL-producing *E. coli* (aOR = 2.09, 95% CI = 1.10–3.98). Additionally, the possession of chicken increased the odds of infection with ESBL-producing *E. coli* five times (aOR = 5.13, 95% CI = 1.66–15.68). The sex and age of the child were not found to be significantly associated with infection with ESBL-producing *E. coli.* Similarly, although owing soap or washing hands with water and soap showed a trend to decrease the risk of contracting ESBL-producing *E. coli,* this association was not statistically significant (Fig. 2).

### Genetic characterization of ESBL strains (conventional PCR)

During PCR screening, we found that the *bla*_CTX-M-15_ gene was the most prevalent resistance gene in both human (82.8%) and animal (100%) isolates. The prevalence of the *bla*_*CTX-M-15*_ resistance gene was similar in isolates from the feces of pastoralists and agro-pastoralists, both almost at 80% (Figure S2 of the Supplementary Materials 1). Comparing the nine isolates from animals alongside those from children residing in the same household, we observed that only two households within the pastoralist group demonstrated simultaneous presence of *bla*_CTX-M-15_ in both their children and livestock (Figure S3).

### Multi locus sequence types (MLST), phylogenetic groups and plasmid MLST

The 48 human *E. coli* isolate subjected to WGS analysis, were chosen based on their phenotypic profile. A sequence type (ST) by MLST using the Achtman scheme could be assigned to 44 isolates (91.7%), with five isolates having a single nucleotide polymorphism (SNP) in one gene (2 in *adk*, 2 in *fumC*, 1 in *mdh*). The most common ST was ST-2353 with five *E. coli* isolates, followed by ST-10 and ST-48 with three *E. coli* isolates each and ST-38, ST-450 and ST-4750 with two *E. coli* isolates each. All other 27 *E. coli* isolates had singular ST. A total of nine *E. coli* isolates were assigned to clonal complex ST10 (3 ST-10, 3 ST-48, 1 ST-227, 1 ST-378 and 1 ST-617) and two isolates to clonal complex ST38 (2 ST-38).

The predominant beta-lactam resistance gene among the 48 *E. coli* isolates was *bla*_CTX-M-15_, identified in 72.9% (35/48) of the whole-genome sequenced isolates, followed by *bla*_TEM-1B_ identified in 47.9% (23/48), ampC beta-lactamase in 14.6% (7/48), *bla*_OXA-1_ in 8.3% (4/48) and *bla*_CTX-M-55_ in 4.2% of the isolates. The beta-lactam resistance genes *bla*_CTX-M-14_ and *bla*_TEM-35_ were the least prevalent, each detected in a single isolate.

Four *E. coli* isolates (8.3%) did not carry any known beta-lactam resistance genes, 20 *E. coli* isolates (41.7%) carried one, 19 *E. coli* isolates (39.6%) carried two and five *E. coli* isolates (10.4%) carried three beta-lactam resistance genes. Seventy-five percent (15/20) of *E. coli* isolates with one beta-lactam resistance gene carried only *bla*_CTX-M-15_, while 68.4% of *E. coli* isolates with two beta-lactam resistance genes carried both *bla*_CTX-M-15_ and *bla*_TEM-1B_.

Quinolone resistance conferred by mutations in the *gyrA* gene was detected in 33.3% (16/48) of *E. coli* isolates. Serine at position 83 was mutated to either Leucine (S83**L**, 10/16), Alanine (S83**A**, 3/16) or Valine (S83**V**, 3/16). In addition to S83**L**, three *E. coli* isolates had *gyrA* mutation D87**N**. Four *E. coli* isolates (8.3%) had *parC* mutation S57**T** and four *E. coli* isolates (8.3%) had *parC* mutation S80**I** combined with *parE* mutation S458**A**. The plasmid-encoded *qnrS1* and *qnrS13* genes were detected in 39.6% (19/48) and 2.1% (1/48) of the sequenced isolates, respectively.

Aminoglycoside resistance genes were present in 60.4% (29/48) of *E. coli* isolates. Four different aminoglycoside (3″) (9) adenylyltransferase (*aadA*) genes were detected in 15 *E. coli* isolates (31.3%), with *aadA1* accounting for 66.7% (10/15), *aadA2* and *aadA24* for 13.3% (2/15) each and *aadA5* for 6.7% (1/15) of the aadA genes found. Eighteen *E. coli* isolates (39.5%) had the plasmid-encoded aph(3″)-Ib and aph(6)-Id aminoglycoside resistance genes, one had only aph(3″)-Ib and one only aph(6)-Id. The aminoglycoside resistance genes aac(3)-IId, aac(6′)-Ib-cr, ant(2″)-Ia and aph(3′)-Ia were detected in one *E. coli* isolate each.

Sulfonamide resistance genes were detected in 62.5% of *E. coli* isolates (30/48), with *sul1* in 30.0% (9/30), *sul2* in 63.3% (19/30) and both genes in 6.7% (2/30) of the isolates. Trimethroprim resistance genes *dfrA* were detected in 58.3% of *E. coli* isolates (28/48), most commonly *dfrA14* (9), followed by *dfrA1* (7), *dfrA5* (3), *dfrA17* (3), *dfrA7* (2), *dfrA15* (2), *dfrA8* (1) and *dfrA19* (1). Tetracycline resistance genes *tetA*, *tetB* and *tetD* were detected in 35.4% (17/48), 16.7% (8/48) and 2.1% (1/48) of *E. coli* isolates, respectively. Macrolide resistance gene *mphA* was detected in 18.8% (9/48) of the isolates, with one isolate simultaneously carrying *ermB*. The results are summarized in figure S4.

### Virulence genes and plasmids

A total of five different virulent genes were found in 15/48 of *E. coli* isolates, but none of the isolates were found to harbor Shigo-toxin genes in their genomes. The results revealed the presence of Enteroaggregative *E. coli* genes such as *aggR* (5/48), *aggA* (2/48) and *aaiC* (2/48). Additionally, both the heat-labile (*elt*) and heat-stable enterotoxigenic *E. coli* genes (*est*) were found in 7/48 and 8/48 *E. coli* isolates (Table S2).

**Fig. 3:**
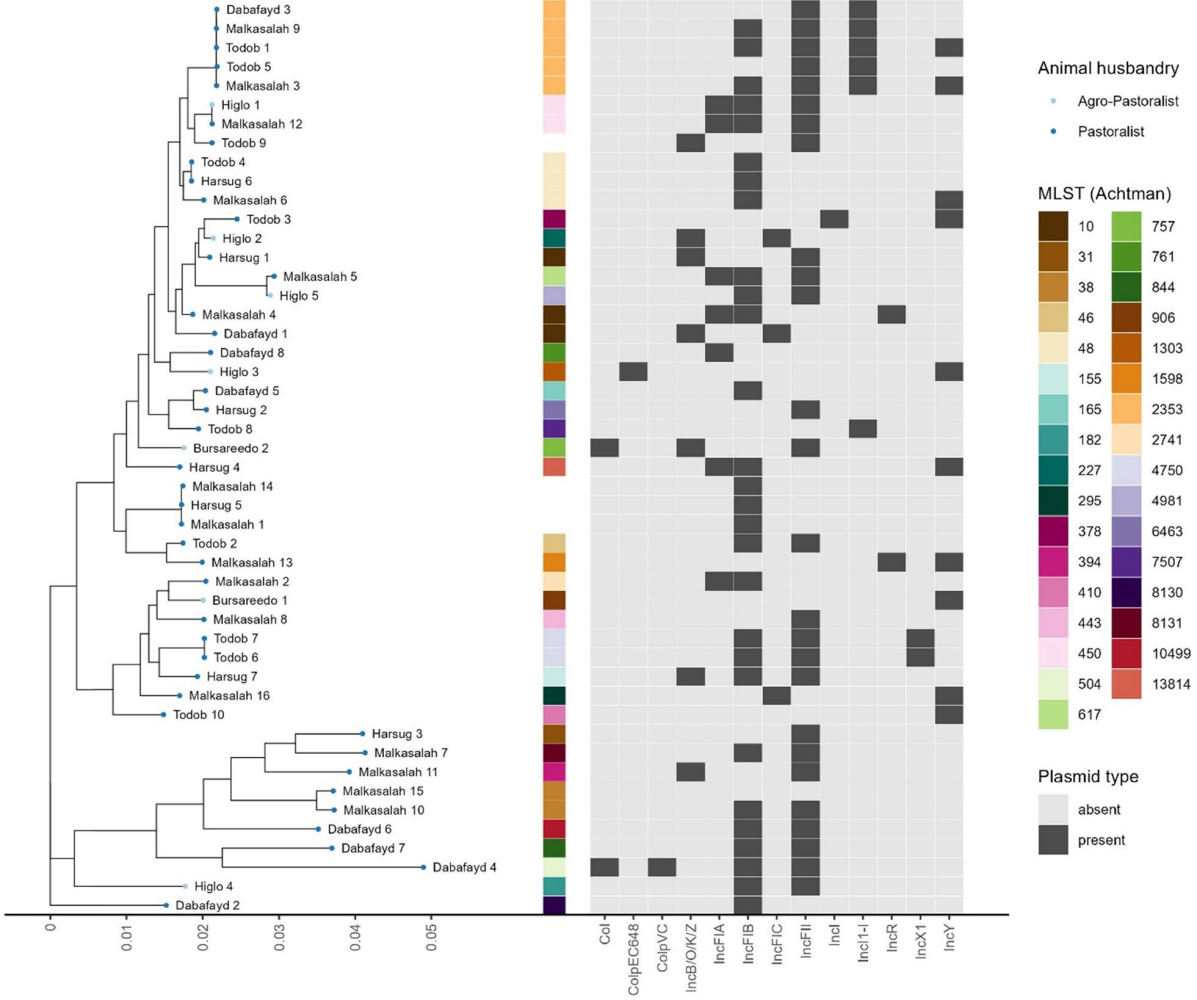
Plasmid replicon profiles for 48 ESBL-producing *E. coli* isolated from 2 to 5 year old children in the Adadle district, Somali Region, Ethiopia. The presence of plasmid is shown in dark gray and absence in light gray

The detection rate of IncFIB plasmid exhibited the highest frequency, found in 28/48 isolates, followed by IncFII (26/48), IncY (10/48), IncFIA (7/48) and Incl1-l (6/48). Combinations of multiple plasmids were observed, with IncFIB and IncFII being the most prevalent combination, present in 17/48 isolates. Specifically, 12 isolates carrying *bla*_*CTX-M-15*_ resistance genes encoded both IncFIB and IncFII plasmids. Three isolates carried a combination of three plasmids (IncFIB, IncFII, IncFIA), and single isolates carried four different plasmids (IncFIB, IncFII, IncFIA, IncY) (Fig. 3).

### Comparison of genotypes and phenotypes

**Fig. 4:**
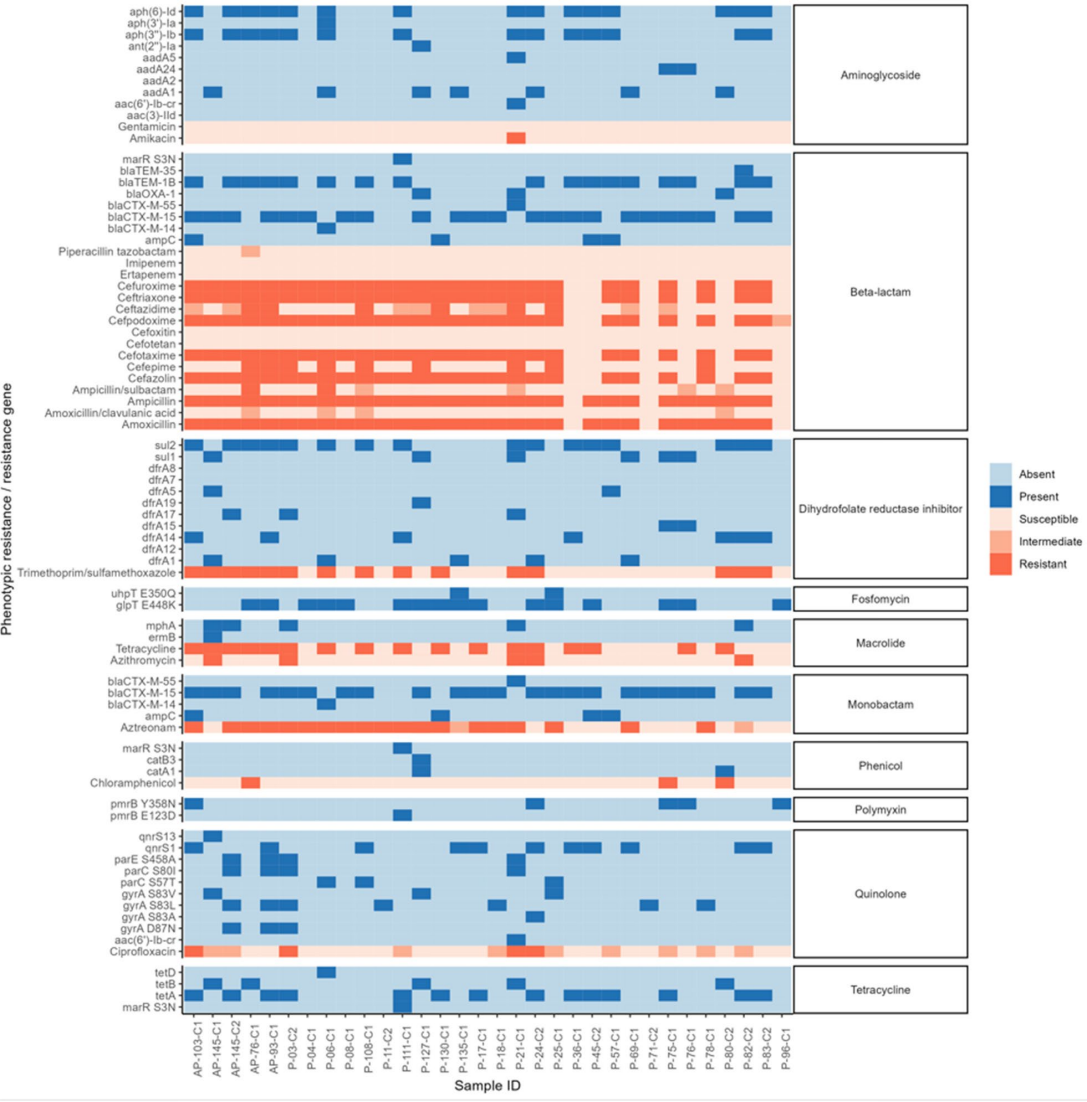
Concordance and discordance between genotype and phenotype for selected ESBL-producing *E. coli* isolates from Adadle district, Somali region, Ethiopia

Resistance to different antibiotics was inferred from WGS data. The presence of specific genes (Fig. 4, in blue) indicated resistance to specific antibiotics (Fig. 4, in red). For example, the presence of at least one of the following genes, CTX-M-type, OXA-type, TEM-type, or ampC, indicated resistance to amoxicillin, ampicillin, aztreonam, cefotaxime, ceftriaxone, cefazolin, cefuroxime, and cefepodoxime. The same procedure was used for all other antibiotics, as indicated in Fig. 4.

The resistance results inferred from WGS data were then compared with the results of the phenotypic assay. We found high concordance rate (> 90%) between genotype and phenotype for azithromycin (93%), amoxicillin (91%), and ampicillin (91%). Conversely, chloramphenicol (87%), as well as several cephalosporins including cefotaxime, ceftriaxone, cefazolin, cefuroxime, and cefepodoxime, each showed a low concordance rate of 84%. Tetracycline (81%) and trimethoprim/sulfamethoxazole (72%) showed even lower concordance rates, with ciprofloxacin (63%) registering the lowest concordance rate among them. These discordances should be analysed in more detail by assessing more antibiotics and by mining for eventual new resistance mechanisms that could be encoded in these bacterial strains. The results are summarized in Fig. 4.

## Discussion

Over the past two decades, there has been a significant increase in the global prevalence of communities carrying ESBL-producing *E. coli* [39]. In LMICs, the colonization of ESBL-producing *E. coli* has risen steadily in both community and healthcare settings, with the community carriage rate approaching that of healthcare settings [40]. To our knowledge, this is the first study to employ WGS for profiling of the phylogenomic of ESBL-producing *E. coli* in children under the age of five in rural communities in Ethiopia.

The results show a high carriage (43%) of ESBL-producing *E. coli* in children under the age of five living in these communities via phenotypic analysis. This finding aligns with a study conducted among hospitalized children in Addis Ababa [23] and rural children in Ghana [41]. The elevated prevalence observed could be attributed to the widespread availability of antimicrobials and misuse of antibiotics [42]. Additionally, in rural communities, limited access to clean water and sanitation may contribute the spread of ESBL-producing *E. coli* resistant clones and genes [43]. On the other hand, low prevalence of ESBL-producing *E. coli* carriage was found in livestock, which aligns with a study performed in Kenya [44]. In rural communities, limited access to veterinary care may result in reduced antibiotic usage in livestock, thereby decreasing the selection pressure for antibiotic-resistant bacteria [45]. This situation could, in turn, contribute to the lower prevalence of ESBL-producing *E. coli* strains in the livestock population.

In the PCR analysis, the resistance gene *bla*_CTX-M-15_ emerged as the most frequently observed gene among *E. coli* isolates that exhibited an ESBL phenotype in our study, both in the children (120/159 isolates) and in livestock (9/9 isolates). Importantly, *bla*_CTX-M-15_ is known as the primary ESBL resistance gene responsible for human infections worldwide [46]. Moreover, recent research conducted in Africa corroborates this finding, highlighting *bla*_CTX-M-15_ as the dominant ESBL-producing *E. coli* gene in livestock populations [47]. This geographical distribution strongly suggests a significant spread of resistance genes among both humans, animals and other domains such as water and food products, highlighting the importance of understanding and addressing this issue from a One Health perspective.

Studies in Ethiopia and Ghana also demonstrated a substantial diversity of STs, accounting for over 30 of them [2, 20]. Here, ST10 and ST48 exhibited multi-drug resistance genes, primarily *bla*_CTX-M-15_ and *bla*_TEM-1B_, along with other non-beta-lactamase resistance genes. Globally, ST10 and ST48 are recognized as clonal genetic entities known to harbor multi-drug resistance genes, primarily in humans, with a notable prevalence of *bla*_CTX-M-15_ [48, 49]. Additionally, ST38 showed a lineage associated with the carriage of *bla*_CTX-M-14_ and *bla*_CTX-M-15_, which is consistent with prior research findings in Tanzania [50, 51].

Notably, ST2353, typically associated with highly pathogenic diarrheagenic *E. coli* strains [52, 53] and historically less reported for its resistance gene carriage, emerged as one of the predominant sequence types in our study. Remarkably, ST2353 was found to harbor multiple resistance genes, encompassing *bla*_CTX-M-15_, *bla*-_TEM-1b_, *gyrA*, and *tetA*. This observation underscores the potential for gene evolution over time, signifying the spread of resistance mechanisms across diverse clonal sequence types. This gene can impact the efficacy of antibiotics treatment on a population level. Hence, our findings underscore the imperative need for further comprehensive investigations aimed at elucidating the mechanisms underlying genetic mutations and the emergence of allelic variants associated with antibiotic resistance.

The IncFII-FIA-FIB multi-replicon plasmids have been commonly associated with *bla*_CTX-M-15_ resistance genes globally [54]. This was also found in our study along with other studies in Ethiopia, Tanzania, and Ghana [21, 55, 56]. These plasmids can maintain and transfer resistance genes among enterobacterial species independent of antibiotic exposure, which contributes to the dissemination of AMR genes with in the community [57]. Therefore, genomic surveillance is essential to tracking the evolution of resistance determinants and assessing the effectiveness of interventions aimed at controlling AMR spread in different ecological settings.

We report a high concordance rate (> 90%) between genotypic and phenotypic resistance for certain antibiotics, as similarly reported by others [58, 59]. However, lower concordance rates (< 90%) were observed for certain antibiotics classes, with fluoroquinolones showing the lowest agreement. Most cases were so-called “major errors”, i.e. genotypic resistance was observed but phenotypic susceptibility was not [60]. This phenomenon may be attributed to the suppression of gene expression through transcriptional regulation, other gene silencing mechanisms or compensatory mutations [61, 62]. Similar results were noted in other studies conducted in Singaopore and France [59, 61]. Further research is needed to fully explain this phenomenon.

In the multivariable analysis, we found that children whose mother were illiterate had higher odds of carrying ESBL-producing *E. coli* [63]. Furthermore, age and sex of the child were not significantly associated with ESBL-producing *E. coli* carriage. These results are consistent with prior studies conducted in Ethiopia, Guinea-Bissau, and Madagascar [63–65]. The observed link between maternal illiteracy and ESBL-producing *E. coli* colonization suggests the importance of community education in implementing effective strategies to combat this public health concern.

Children who were both stunted and wasted were significantly more likely to be colonized by ESBL-producing *E. coli*, with a three times higher odds compared with those without malnutrition [66–68]. This finding can be explained by the fact that malnutrition weakens the immunity system, making children more susceptible to infections, and more likely to be treated with antibiotics [69]. This connection may also involve the influence of microbiota and the creation of niches for colonization. Given the well-established link between antibiotic use and the emergence and spread of AMR, addressing malnutrition through public health interventions could contribute to the reduction of infections and colonization by AMR carrying bacteria.

Furthermore, the study revealed that there was a significant correlation between ESBL carriage and settlement type, water treatment, and chicken ownership. Pastoralists were found to have higher odds of ESBL-producing *E. coli* colonization compared to agro-pastoralists. The nomadic nature of pastoralists, involving frequent travels in search of water and food, may foster shared water sources and inadequate sanitation and hygiene practices. Consequently, this situation could escalate the risk of waterborne diseases like diarrhea and facilitate the transmission of ESBL-producing *E. coli* [70]. As infectious diseases become more prevalent in such contexts, there is an associated rise in antibiotic usage, a well-established precursor to AMR [71].

Despite chlorine being the most commonly used method for water treatment in LMICs [72], this study found a positive association between children who consumed chlorine-treated water and ESBL-producing *E. coli*. This finding aligns with a randomized control trial conducted in Bangladesh, which revealed that water chlorination did not significantly decrease the fecal carriage of ESBL-producing *E. coli* in children [73]. Other studies conducted in China and South Africa also demonstrated the tolerance of chlorine in relation to AMR [74, 75]. Furthermore, a study conducted in China highlighted that chlorination promotes horizontal gene transfer through natural transformation, thus facilitating the spread and emergence of AMR [76]. Therefore, we hypothesize that the widespread use of chlorine in LMICs for water treatment may not be as effective as previously thought in reducing the prevalence and transmission of antimicrobial-resistant *E. coli* strains.

In this study, possessing chickens showed a significant relationship with ESBL-producing *E. coli*, with the odds of colonization being five times higher. Previous studies conducted in Ethiopia, Kenya, Nigeria, Ghana, Pakistan, and the Netherlands have suggested that chickens are the primary source of ESBL-producing *E. coli* transmission to humans [2, 77–82]. This suggests that there is a need of effective measures to control the spread of antimicrobial resistance in animal husbandry, with a focus on poultry as the main carriers.

We attempted to collect fecal samples from children and livestock within the same households to investigate the circulation of resistance genes between the two populations. However, due to the absence of necessary kits and machines for whole genome sequencing (WGS), we were constrained to ship the DNA to Switzerland to carry out the WGS. Since the shipment was costly, we were only able to send the DNA from human and animal fecal samples, as well as the original human fecal samples.

After running a set of DNA isolates from both human and animal samples on Nanopore, we discovered that the DNA was fragmented. Fortunately, because the human fecal samples had been shipped, we were able to re-isolate and re-extract the DNA, and successfully perform WGS. However, since we did not have the animal fecal samples in Switzerland, we were unable to conduct WGS on these samples. This limitation restricted our ability to explore the genetic aspects of resistance gene transmission between human and animal populations comprehensively. Additionally, the limited sequencing data derived from human fecal isolates, along with the absence of sequenced data from animal fecal isolates and other One Health domains, has narrowed our focus and restricted our ability to conduct comprehensive analysis of mobile genetic elements, such as phages and insertion sequence, which is essential for the dissemination of an antimicrobial resistance. Instead of, we anticipate addressing this limitation in future projects by including a broader range of samples. This will enable a more comprehensive analysis and will contribute to the understanding of transmission and dissemination of AMR in different domains of One Health.

## Conclusion

Our study represents the first study of molecular epidemiology of ESBL-producing *E. coli* isolated from rural children and livestock in Ethiopia. We found high and low prevalence of ESBL-producing *E. coli* in rural children and livestock, respectively, largely mediated by the gene *bla*_CTX-M-15_ encoded on a plasmid. The isolates displayed a high diversity of STs, with the predominant types being ST-2353, ST-10, ST-48, ST-38, and ST-450. Our study is the first to report that ST-2353 is associated with multi-drug resistance genes in Ethiopia. Further research including other domains such water and food products are needed to comprehensively study this diversity and the spread of antimicrobial resistance genes to better understand their acquisition. We suggest implementing an integrated One Health surveillance system, which would be able to monitor transmission events and detect resistant bacteria in a timely manner from both humans and animals.

## Supplementary Information

Additional file1 (DOCX 456 KB)

## Data Availability

No datasets were generated or analysed during the current study.

## Abbreviations

AMR: Antimirobial resitance
ESBL: Extended spectrum beta lactamase
*E. coli*: *Eschericia coli*
WGS: Whole genome sequencing
MLST: Multilocus sequence typing
SRS: Somali regional state
