# Supplementary material for: Fecal carriage of ESBL-producing E. coli and genetic characterization in rural children and livestock in the Somali region, Ethiopia: a one health approach
Source: Antimicrob Resist Infect Control. 2024 Dec 18;13:148. doi: 10.1186/s13756-024-01502-5 (PMC11656975; doi:10.1186/s13756-024-01502-5)
Supplement: Supplementary file 1 — Additional file1 (DOCX 456 KB) [file 13756_2024_1502_MOESM1_ESM.docx]

**Results**

Table S1: Household WASH Characteristics and Livestock Ownership among Pastoralist and Agro-pastoralist Communities in Adadle District, Somali Region, Ethiopia

| **Variables** | **Agro-Pastoralist**, N = 169 | **Pastoralist**, N = 177 | **Overall**, N = 346 |
| --- | --- | --- | --- |
| **Water Source** |  |  |  |
| Birkad /Borehole | 3 (1.8%) | 157 (88.7%) | 160 (46.2%) |
| River water | 161 (95.3%) | 5 (2.8%) | 166 (48%) |
| Tank truck | 5 (3.0%) | 15 (8.5%) | 20 (5.8%) |
| **Treat water** |  |  |  |
| No | 97 (57.4%) | 155 (87.6%) | 252 (72.8%) |
| Yes | 72 (42.6%) | 22 (12.4%) | 94 (27.2%) |
| **Toilet type** |  |  |  |
| Outdoor | 168 (99%) | 164 (93%) | 332 (96%) |
| Pit latrine | 1 (0.6%) | 13 (7.3%) | 14 (4.0%) |
| **Shared toilet** |  |  |  |
| No | 151 (89.3%) | 167 (94.4%) | 318 (91.9%) |
| Yes | 18 (10.7%) | 10 (5.6%) | 28 (8.1%) |
| **Waste disposal** |  |  |  |
| Burned | 25 (14.8%) | 29 (16.4%) | 54 (15.6%) |
| Dumped in the street/open space | 144 (85.2%) | 148 (83.6%) | 292 (84.4%) |
| **Hand washing method** |  |  |  |
| Water and soap | 15 (8.9%) | 5 (2.8%) | 20 (5.8%) |
| With water only | 154 (91.1%) | 172 (97.2%) | 326 (94.2%) |
| **Household had soap** |  |  |  |
| No | 31 (18.3%) | 15 (8.5%) | 46 (13.3%) |
| Yes | 95 (56.2%) | 103 (58.2%) | 198 (57.2%) |
| Sometimes | 43 (25.4%) | 59 (33.3%) | 102 (29.5%) |
| **Cattle** |  |  |  |
| No | 9 (5.3%) | 124 (70.1%) | 133 (38.4%) |
| Yes | 160 (94.7%) | 53 (29.9%) | 213 (61.6%) |
| **Camel** |  |  |  |
| No | 152 (89.9%) | 113 (63.8%) | 265 (76.6%) |
| Yes | 17 (10.1%) | 64 (36.2%) | 81 (23.4%) |
| **Chicken** |  |  |  |
| No | 151 (89.3%) | 177 (100.0%) | 328 (94.8%) |
| Yes | 18 (10.7%) | 0 (0.0%) | 18 (5.2%) |
| **Goat** |  |  |  |
| No | 63 (37.3%) | 22 (12.4%) | 85 (24.6%) |
| Yes | 106 (62.7%) | 155 (87.6%) | 261 (75.4%) |
| **Sheep** |  |  |  |
| No | 79 (46.7%) | 99 (55.9%) | 178 (51.4%) |
| Yes | 90 (53.3%) | 78 (44.1%) | 168 (48.6%) |
| **Donkey** |  |  |  |
| No | 32 (18.9%) | 73 (41.2%) | 105 (30.3%) |
| Yes | 137 (81.1%) | 104 (58.8%) | 241 (69.7%) |


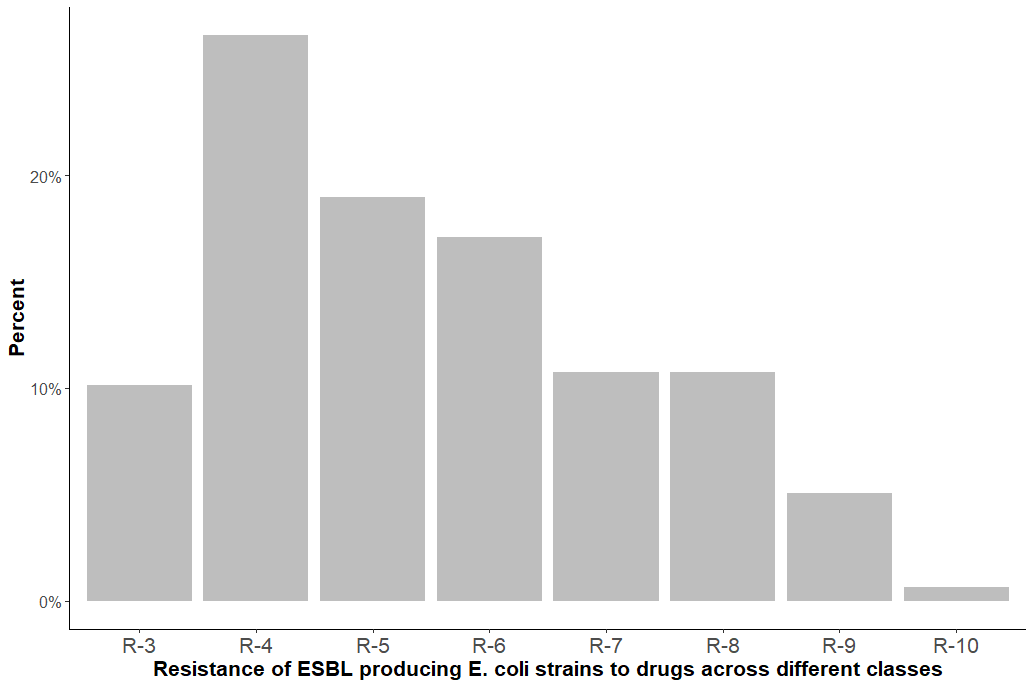


Figure S1: Multidrug resistance pattern in ESBL-producing *E. coli* among children in Adadle district Somali region, Ethiopia. R-3: Resistance to three drugs from different classes; R-4: Resistance to four drugs from different classes; R-5: Resistance to five drugs from different classes; R-6: Resistance to six drugs from different classes; R-7: Resistance to seven drugs from different classes; R-8: Resistance to eight drugs from different classes; R-9: Resistance to nine drugs from different classes; R-10: Resistance to ten drugs from different classes.


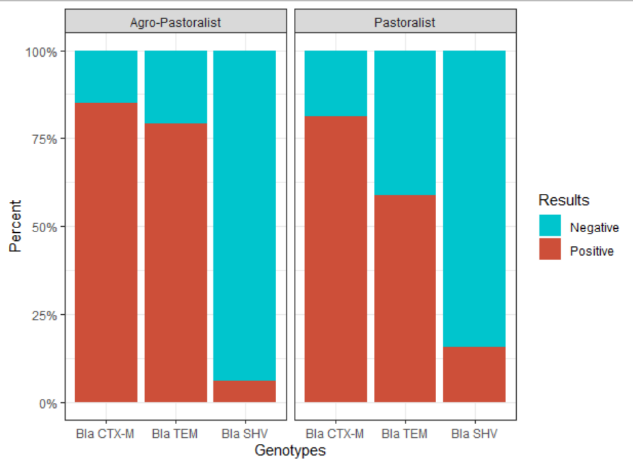


Figure S2: Characterization of the resistance genes in ESBL-producing *E. coli* among children in Adadle district, Somali region, Ethiopia


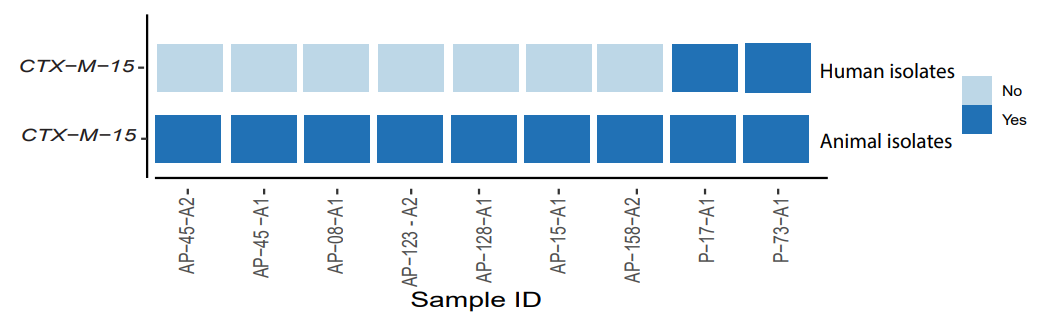


Figure S3: Comparing resistance gene of all animal isolates and at the same time isolates from children that live in the same household from Adadle district, Somali region, Ethiopia


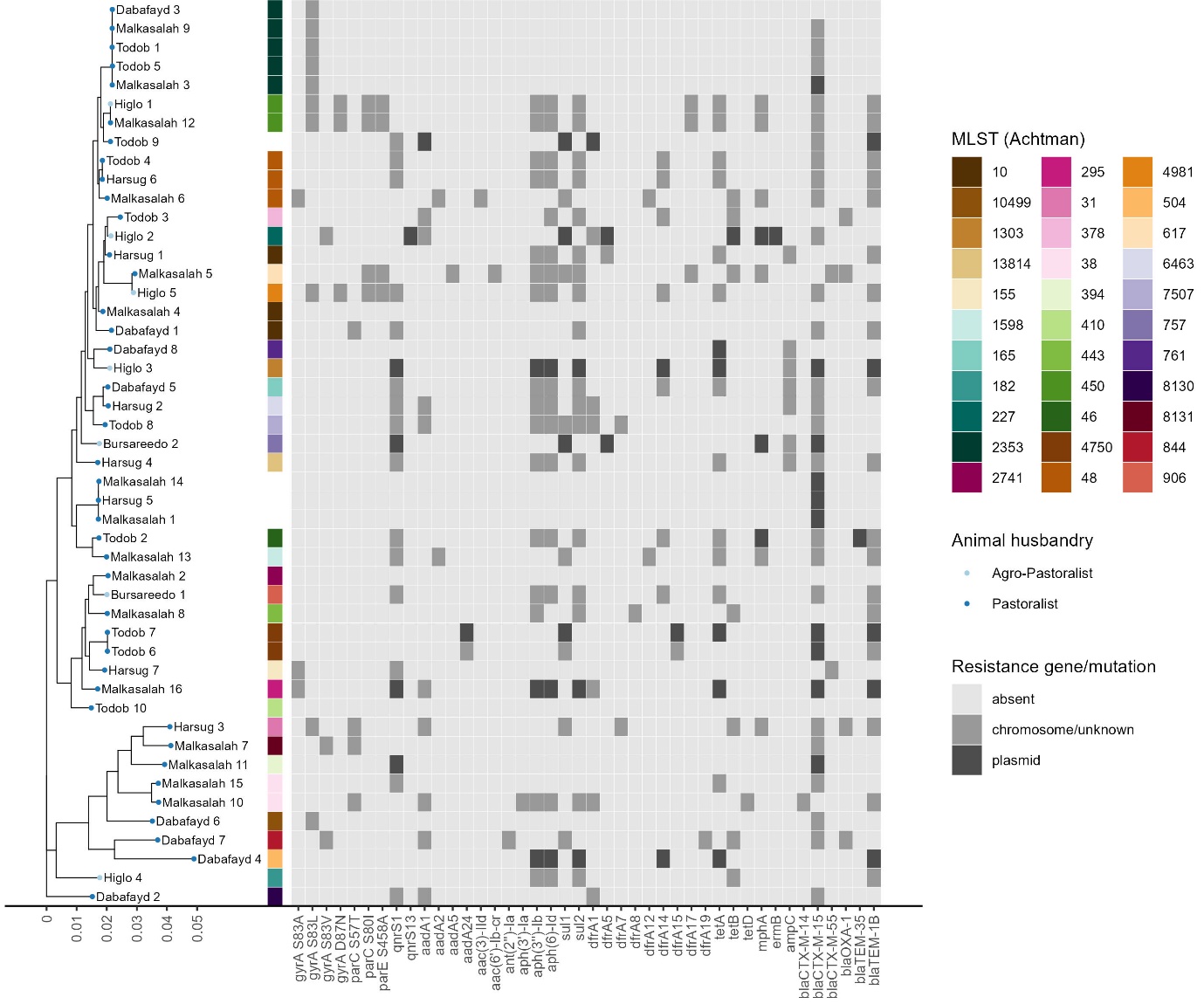


Figure S4: Phylogenetic analysis of AMR markers and MLST based sequence types in 48 ESBL-producing *E. coli* isolated from 2-5 year old children in the Adadle *woreda*, Somali Region, Ethiopia. The presence of AMR markers is shown in light gray if located in the genome or an unidentified plasmid and dark gray if located on a plasmid. MLST are indicated with the color code indicated on the right of the figure.

Table S2: Plasmid types and virulent genes from ESBL-producing *E. coli* isolates among children in Adadle district, Somali region, Ethiopia

| **Isolate code** | **Plasmid type** | **Virulence genes** | **Accessation number** |
| --- | --- | --- | --- |
| Dabafayd 2 | IncFIB |  | SAMN41910778 |
| Higlo 4 | IncFIB, IncFII | *STa* | SAMN41910792 |
| Dabafayd 4 | IncFIB, IncFII, col, colpVC |  | SAMN41910788 |
| Dabafayd 7 | IncFIB, IncFII | *elt* | SAMN41910796 |
| Dabafayd 6 | IncFIB, IncFII |  | SAMN41910795 |
| Malkasalah 10 | IncFIB, IncFII | *aggA, aggR* | SAMN41910797 |
| Malkasalah 15 |  |  | SAMN41910807 |
| Malkasalah 11 | IncFII, IncB |  | SAMN41910800 |
| Malkasalah 7 | IncFIB, IncFII | *aggR, aggA* | SAMN41910782 |
| Harsug 3 | IncFII | *aaiC, aggR* | SAMN41910787 |
| Todob 10 | IncY |  | SAMN41910812 |
| Malkasalah 16 | IncFIC, IncY | *aaiC, aggR* | SAMN41910810 |
| Harsug 7 | IncFIB, IncFII, IncB | *elt, STa* | SAMN41910809 |
| Todob 6 | IncFIB, IncFII, IncX1 |  | SAMN41910791 |
| Todob 7 | IncFIB, IncFII, IncX1 |  | SAMN41910794 |
| Malkasalah 8 | IncFII | *STa* | SAMN41910783 |
| Bursaredo 1 | IncY |  | SAMN41910768 |
| Malkasalah 2 | IncFIB, IncFIA |  | SAMN41910771 |
| Malkasalah 13 | IncR, IncY |  | SAMN41910804 |
| Todob 2 | IncFIB, IncFII |  | SAMN41910776 |
| Malkasalah 1 | IncFIB |  | SAMN41910769 |
| Harsug 5 | IncFIB |  | SAMN41910802 |
| Malkasalah 14 | IncFIB |  | SAMN41910805 |
| Harsug 4 | IncFIA, IncFIB, INcY |  | SAMN41910799 |
| Bursareedo 2 | IncB, IncFII, Col |  | SAMN41910784 |
| Todob 8 | Incl1-l |  | SAMN41910798 |
| Harsug 2 | IncFII |  | SAMN41910770 |
| Dabafayd 5 | IncFIB |  | SAMN41910793 |
| Higlo 3 | ColpEC648, IncY |  | SAMN41910785 |
| Dabafayd 8 | IncFIA |  | SAMN41910811 |
| Dabafayd 1 | IncFIC, IncB |  | SAMN41910777 |
| Malkasalah 4 | IncFIB, IncFIA, IncR |  | SAMN41910773 |
| Higlo 5 | IncFIB, IncFII |  | SAMN41910806 |
| Malkasalah 5 | IncFIB, IncFII, IncFIA |  | SAMN41910774 |
| Harsug 1 | IncFII, IncB |  | SAMN41910766 |
| Higlo 2 | IncFIC, IncB | *aggR* | SAMN41910767 |
| Todob 3 | Incl, IncY |  | SAMN41910781 |
| Malkasalah 6 | IncFIB, incY |  | SAMN41910780 |
| Harsug 6 | IncFIB |  | SAMN41910808 |
| Todob 4 | IncFIB |  | SAMN41910786 |
| Todob 9 | IncFII, IncB |  | SAMN41910801 |
| Malkasalah 12 | IncFIA, IncFIB, IncFII |  | SAMN41910803 |
| Higlo 1 | IncFIA, IncFIB, IncFII |  | SAMN41910765 |
| Malkasalah 3 | IncFIA, IncFIB, IncFII, IncY | *elt, STa* | SAMN41910772 |
| Todob 5 | IncFIB, IncFII | *STa, elt* | SAMN41910789 |
| Todob 1 | IncFIA, IncFIB, IncFII, IncY | *elt, STa* | SAMN41910775 |
| Malkasalah 9 | IncFIA, IncFIB, IncFII | *elt, STa* | SAMN41910790 |
| Dabafayd 3 | IncFIB, IncFII | *elt, STa* | SAMN41910779 |

**Methods and Materials**

**Molecular testing for β-lactamase genes (conventional PCR)**

Table S3: Primer Sequences for PCR amplifications for ESBL genes

| Target gene | Oligo name | Oligo Sequence 5’ and 3’ | Size (bp) | Reference |
| --- | --- | --- | --- | --- |
| *bla*CTX-M | CTX-M-F | CGCTGTTGTTAGGAAGTGT | 754 | [1] |
|  | CTX-M-R | GGCTGGGTGAAGTAAGTGA |  |  |
| *bla*TEM | TEM-F | TTTCGTGTCGCCCTTATTC | 404 | [2] |
|  | TEM-R | ATCGTTGTCAGAAGTAAGTTG |  |  |
| *bla*SHV | SHV-F | CGCCTGTGTATTATCTCCC | 294 | [2] |
|  | SHV-R | CGAGTAGTCCACCAGATCC |  |  |

**PCR conditions**

The reaction comprised 12.5 μL of 2X HotStartTaq multiplex PCR Master Mix (QIAGEN), 1.5 μL of each primer (forward and reverse, 0.2 μM), 1.5 μL of template DNA (300 ng), and 9.5 μL of nuclease-free water.

PCR cycling conditions comprised an initial denaturation step at 95^o^C for 15 minutes, followed by 35 cycles of denaturation for 30 seconds, annealing at 58^o^C for 90 seconds, and extension at 72^o^C for 90 seconds. A final elongation step was carried out at 72^o^C for 10 minutes. The results were visualized through gel electrophoresis S3.

**Selection criteria for the isolates that were subjected to whole genome sequencing**

In this study, the initial set of analyses and DNA extraction was performed in Ethiopia. Nano-drop was used to assess the quality of the DNA samples as this was the only DNA quantification machine available on site, as we did not have the kit for the Qubit analysis. Subsequently, the extracted DNA from human and animal ESBL-producing isolates were shipped to the Swiss Tropical and Public Health Institute (Swiss TPH) for whole genome sequencing (WGS). Prior to sequencing, we subjected the extracts to Qubit analysis to more accurately measure DNA concentration. We then concentrated the DNA using the Zymo DNA Clean & Concentrator kit, and finally performed gel electrophoresis on QIAxcel (Qiagen) to check the fragment size of the DNA. The DNA quality of the sub-isolates, selected based on their phenotypic profiles, was insufficient for WGS. Since we did not have the original isolates in Switzerland, we decided to re-culture and perform DNA extraction on the original fecal samples. The fecal samples included in this *de novo* strain isolation were originally selected based on the phenotypic profiles obtained in Ethiopia. Due to this *de novo* isolation, PCR and WGS data is provided on strains isolated from the same fecal sample, that are however not necessarily the same strains. Both set of strains were tested for their phenotypic resistances.

**Reference**

1. Ramachandran, A., M. Shanthi, and U. Sekar, *Detection of bla(CTX-M) Extended Spectrum Beta-lactamase Producing Salmonella enterica Serotype Typhi in a Tertiary Care Centre.* J Clin Diagn Res, 2017. **11**(9): p. Dc21-dc24 DOI: 10.7860/jcdr/2017/30150.10637 ISSN: 2249-782X.

2. Mohammed, Y., et al., *Characterization of Extended-Spectrum Beta-lactamase from Escherichia coli and Klebsiella Species from North Eastern Nigeria.* J Clin Diagn Res, 2016. **10**(2): p. Dc07-10 DOI: 10.7860/jcdr/2016/16330.7254 ISSN: 2249-782X.
